# Performance of Loop-Mediated Isothermal Amplification Technique in Milk Samples for the Diagnosis of Bovine Tuberculosis in Dairy Cattle Using a Bayesian Approach

**DOI:** 10.3390/pathogens11050573

**Published:** 2022-05-12

**Authors:** Tawatchai Singhla, Surachai Pikulkaew, Sukolrat Boonyayatra

**Affiliations:** Department of Food Animal Clinic, Faculty of Veterinary Medicine, Chiang Mai University, Chiang Mai 50100, Thailand; tawatchai.singh@cmu.ac.th (T.S.); surachai.pikul@cmu.ac.th (S.P.)

**Keywords:** bovine tuberculosis, loop-mediated isothermal amplification, single intradermal tuberculin test, Bayesian modeling, test performance

## Abstract

This study aimed to estimate the sensitivity (Se) and specificity (Sp) of loop-mediated isothermal amplification (LAMP) and single intradermal tuberculin (SIT) tests for the diagnosis of bovine tuberculosis (bTB) in dairy cattle in Thailand using a Bayesian approach. The SIT test was performed in 203 lactating dairy cattle from nine dairy farms located in Chiang Mai province, Thailand. Milk samples were collected for the LAMP test. Kappa analysis was performed to determine the agreement between the two tests. A one-population conditional independence Bayesian model was applied to estimate the Se and Sp of the two tests. Of 203 dairy cattle, 2 were positive for the SIT test using standard interpretation, whereas 38 were positive for the LAMP test. A poor agreement (kappa = 0) was observed between the two tests. The median Se and Sp of the SIT test using standard interpretation were 63.5% and 99.1%, respectively. The median Se and Sp of the LAMP test were 67.2% and 82.0%, respectively. The estimated true prevalence of bTB was 3.7%. The LAMP test with milk samples can potentially be used as a non-invasive screening test for the diagnosis of bTB in dairy cattle.

## 1. Introduction

Bovine tuberculosis (bTB) is caused by *Mycobacterium bovis* (*M. bovis*), which is a member of the *Mycobacterium tuberculosis* complex. Domestic and wild animals, especially beef and dairy cattle, can also be infected with the bacterium. Moreover, *M. bovis* can be transmitted to humans as a neglected zoonotic disease, and it can result in economic losses worldwide [1]. The World Health Organization reported that the number of new cases of human zoonotic tuberculosis caused by *M. bovis* in 2017 was 142,000, with over 12,500 deaths, mainly in Africa and Southeast Asia [2]. It has been estimated that bTB causes annual losses of approximately USD 3 billion in the livestock economy worldwide [3].

The success of the bTB eradication program depends on the accuracy of the diagnostic test and removal of infected animals. Single intradermal tuberculin (SIT) tests are generally used for bTB diagnosis in live animals in many countries. The SIT test is based on the detection of the cell-mediated immune (CMI) response and is performed by inoculating bovine purified protein derivative (PPD) into the skin of animals. The test results are interpreted by measuring the difference in skin thickness before and after inoculation [4]. This technique is easy and inexpensive to perform. However, the SIT test is a laborious technique, and its accuracy varies with sensitivity (Se) and specificity (Sp) with ranges of 43.2–96.8% and 82.9–99.0%, respectively [4,5]. Moreover, the test results can be influenced by cross-reactions with other mycobacteria and host response variation [6]. 

Molecular techniques have been increasingly used to enhance the accuracy of bTB diagnoses [7]. Polymerase chain reaction (PCR) is a fast and highly sensitive technique that has been widely used for bTB detection. However, this technique should be performed as a post-mortem diagnostic test and requires a well-equipped laboratory [8]. Loop-mediated isothermal amplification (LAMP) is another method developed for the diagnosis of several diseases in animals, including brucellosis and leptospirosis [9,10]. The LAMP technique does not require expensive equipment. Moreover, the procedure is rapid and easy to perform with incubation at a constant temperature. This method could detect *M. bovis* genomic DNA as low as 10 copies within 40 min [11]. Several studies have reported the development of LAMP for the detection of pathogens causing various diseases in bovine milk, such as brucellosis and mycoplasma mastitis [9,12]. However, the performance of this method in the detection of *M. bovis* in raw milk has not been assessed.

The accuracy of a diagnostic test is generally evaluated by comparing the results with those of a gold standard or a reference test. However, in many situations, the results of the gold standard are unavailable. To address this problem, Bayesian latent class modeling can be used to determine the efficacy of a diagnostic test. The Bayesian approach has been increasingly applied to many novel diagnostic tests for various diseases [13,14,15]. Therefore, this study aimed to estimate the Se and Sp of LAMP performed in raw milk and SIT tests using a Bayesian approach.

## 2. Results

### 2.1. bTB Detection Using SIT and LAMP Tests

Of 203 dairy cows, 2 (1%) and 13 (6.4%) were SIT-positive using the standard and severe interpretations, respectively. Compared with the SIT test, a higher proportion of positive results was detected among the study animals using the LAMP test (38/203, 18.7%) (Table 1). The agreement between the LAMP test and the SIT test using both standard (kappa = 0) and severe interpretations (kappa = 0.13) was poor. 

### 2.2. Test Performance of SIT and LAMP Tests

The posterior estimates for the Se of the SIT test were 63.5% (95% posterior probability interval (PPI) = 42.1–81.9%) and 76.1% (95% PPI = 55.7–90.9%) when standard and severe interpretations were applied, respectively. The posterior estimates of the Se of the SIT were lower than the prior estimates. The posterior estimates for the Sp of the SIT test were similar to their prior estimates, regardless of the standard (99.1%) and severe interpretations (96.0%) (Table 2 and Table 3).

The posterior estimates for the Se of the LAMP test were 67.2% (95% PPI = 40.5–88.4%) and 68.8% (95% PPI = 44.8–88.9%) when analyzed in the models with the standard and severe interpretations of the SIT test results, respectively. The posterior estimates for the Sp of the LAMP test were 82.0% (95% PPI = 76.1–87.1%) and 84.1% (95% PPI = 78.2–89.2%) when analyzed in the models with the standard and severe interpretations of the SIT test results, respectively. The posterior estimates of the Sp in the LAMP test were lower than the prior estimates.

The posterior estimates of the true prevalence of bTB in dairy cattle were lower than the prior estimates and varied depending on the interpretation criteria used for the SIT test results, with median values ranging from 1.4% (standard interpretation) to 12.1% (severe interpretation) (Table 2 and Table 3, respectively).

After a visual inspection of the Gelman–Rubin diagnostic plot, the final model converged properly, and autocorrelation was eliminated after omission of the first 10,000 iterations. For the sensitivity analyses, there was no appreciable effect (change > 25% of the median value) in the posterior estimates for the Se of the LAMP test and the Sp of both tests when non-informative distributions were used as priors for any parameter. This finding is interpreted as evidence of the robustness of the model. However, a larger change in the posterior estimates for the SIT test using the standard interpretation (67.2%–26.2%) and severe interpretation (76.1%–35.3%) was observed. Similarly, the prevalence estimates of bTB in the dairy cattle population decreased from 3.7% to 0.7% for the SIT test using standard interpretation and from 6.7% to 4.6% for the SIT test using severe interpretation when a non-informative prior was used. Therefore, these results suggest that these parameter priors have strong effects on the model.

## 3. Discussion

The present study assessed the performance of a bTB screening test routinely used in the national bTB control program (SIT test) in Thailand and an alternative molecular technique (LAMP test) performed in milk samples using a Bayesian approach. A one-population model was chosen for the analysis because the screening tests were performed in infected dairy herds located in the same region and with similar management practices. Therefore, considering all dairy cattle as a single population is reasonable, as assumed in previous studies [5,16].

In general, the main route of infection of *M. bovis* in cattle is via inhalation. Therefore, bTB lesions are mostly found in the respiratory tract, such as the lungs and associated lymph nodes. However, the pathogen can spread via lymphatic ducts and enter other lymph nodes, such as mesenteric and supramammary lymph nodes, which consequently causes the presence of *M. bovis* in bovine milk [1]. The current study reported a higher bTB detection rate when the LAMP technique was used to detect *M. bovis* DNA in milk than when the SIT test was used. A study in India reported the successful isolation of *M. bovis* from milk samples of cows [17]. Moreover, a study in Brazil suggested the detection of *M. bovis* in the milk samples of cattle with negative results of intradermal tuberculin tests when multiplex PCR was applied [18]. These previous studies, together with the results from the present study, confirm that *M. bovis* can contaminate the milk of infected cows, even though some of them showed negative reactions to the intradermal tuberculin test. This phenomenon might explain the disagreement between the LAMP and SIT tests observed in the current study. 

SIT tests are generally performed to control or eradicate bTB worldwide. The test performance indicated that Se and Sp can differ between standard and severe interpretations. Using the standard interpretation, the current study reported the Se of SIT to be 63.5%, which is similar to previous findings in Australia (63.2%) [19] and Thailand (62.4%) [5]. When a severe interpretation was used for the SIT test, a higher Se of 76.1% was estimated in the present study. This finding is similar to a study in Spain reporting the Se of the SIT test to be 69.4% for severe interpretation and 56.6% for standard interpretation [16]. In contrast to Se, the Sp of the SIT test was reported to be high: 99.1% for the standard interpretation and 96% for the severe interpretation. This finding is similar to results previously reported in low-prevalence areas, which range from 83.6% to 100% [16,19]. Both the size of the skin test response and pathological lesions are positively associated with the stage of infection [4,20]. The test-and-slaughter policy has been implemented in Thai dairy cattle for a decade. In some areas, SIT reactors have been continually removed from infected herds. Therefore, animals with advanced stages of infection are rare in dairy herds. This could decrease the Se and increase the Sp of the SIT test when the standard interpretation (inconclusive results defined as negative) is used. 

Using a Bayesian approach, the Se estimates of the LAMP test (67.2–68.8%) agree with the Se of the SIT test. However, the Sp estimates of the LAMP test (82–84.1%) have been reported to be lower than those of the SIT test. A previous study reported that the LAMP test had a high Se and yielded 100% positive results when the test was performed on samples from SIT-positive animals [21]. Moreover, Zhang et al. demonstrated that the LAMP test can detect the DNA of *M. bovis* without any cross-reaction with other mycobacterial DNA, indicating a high Sp of the test [22]. However, the performance of the LAMP test may be affected by the high concentration of calcium ions in milk, which can competitively bind to DNA polymerases with magnesium ions, a necessary cofactor for the reaction [23]. Additionally, prior estimates of disease prevalence given in the current study are based on the reports of previous surveys using only the SIT test. Therefore, the prior estimate of disease prevalence could potentially be underestimated, which could influence the estimation of Se and Sp in both tests.

Currently, the national bTB eradication program using the test-and-cull policy has been implemented in dairy cattle in Thailand. However, administrating SIT to all adult dairy cattle in all herds every year is very labor intensive, costly, and time consuming. Based on our findings, we suggest the use of the LAMP technique as a screening test performed with individual cow’s milk prior to the SIT. For those cattle whose milk samples are LAMP-positive, they should be considered as infected animals and removed from the herd without performing SIT. This strategy can reduce the number of animals required to be tested with SIT by testing only lactating cows with negative LAMP results and non-lactating animals. Moreover, the application of SIT has a limitation on the frequency of testing. In general, if an animal is injected with bovine PPD, that particular animal should not be re-tested with SIT within 60 days after the last injection [24,25,26]. It has been well described that repeated SIT testing in animals infected with *M. bovis* within a period shorter than 60 days might result in a false-negative result due to the desensitization during this period [24]. Therefore, the progress on the eradication of bTB from an infected herd can be prolonged. In contrast, LAMP with milk samples can be performed many times without limitation. In short, the use of LAMP can potentially improve the progress and success of the bTB control and eradication programs in the country.

## 4. Materials and Methods

### 4.1. Study Population

The study was conducted between April and October, 2016. Nine dairy herds with a history of SIT-positive cattle from the Chiang Mai province of Thailand were selected for this study. These herds were previously considered to be bTB-infected based on the presence of at least one SIT-positive animal on the farm during 2011–2015. These farms were smallholder dairy farms with 20–60 lactating cows. Dairy cattle raised on these farms were mixed Holstein Friesian. Three farms used tied stalls, whereas the other six farms adopted free stalls for farm management. 

This study was approved by the Animal Use Ethics Committee of the Faculty of Veterinary Medicine at Chiang Mai University (S30/2559). All farm owners were informed of the study and provided consent before participation.

### 4.2. SIT Test

The caudal fold SIT test was performed by the staff of the Thai Department of Livestock Development as part of the regular annual testing of bTB in dairy cattle. Briefly, bovine PPD (Bovituber^®^ PPD, Synbiotics, Lyon, France) was applied to all adult dairy cattle (age > 1 year) in each herd. The caudal-fold SIT test was performed on dairy cattle by an intradermal injection of 0.1 mL of bovine PPD (2000 IU). The skin thickness at the inoculation site was measured pre- and 72 h post-injection using calipers. Interpretations of the test results were performed according to the Thai agricultural standard for screening tests for bTB [27]. The results were defined as positive when the increase in the skinfold thickness at the inoculation site was >5 mm and/or signs of swelling, edema, exudation, necrosis, and/or inflammation were observed; inconclusive when the increase in the skinfold thickness was 2–5 mm and clinical signs at the inoculation site were not observed; and negative when the skinfold thickness increased by <2 mm and clinical lesions at the injection site were not observed. Depending on the interpretation used, inconclusive animals were considered positive (severe interpretation) or negative (standard interpretation) for the data analysis.

### 4.3. Milk Sample Collection

A total of 203 lactating dairy cows from all nine herds were randomly selected for the study. A composite milk sample from each cow was aseptically collected as described by the National Mastitis Council [28]. Briefly, the udders and teats of the cows were washed and dried. Several streams of foremilk were discarded from all four quarters. The teat end was scrubbed using a cotton ball soaked in 70% alcohol. Milk samples were collected from all quarters into the same labeled test tube. Milk samples were kept in an icebox and transferred to the Central Laboratory of the Faculty of Veterinary Medicine, Chiang Mai University.

### 4.4. Genomic DNA Extraction from Milk

Genomic DNA was extracted from the milk samples using the NucleoSpin^®^ Tissue kit (MACHEREY-NAGEL GmbH&KG, Düren, Germany). Briefly, 10 mL of the milk sample was centrifuged at 8000× *g* for 5 min. The supernatant was discarded, and the pellet was added to 180 µL of buffer T1. Then, 25 µL of proteinase K was added to the pellet. The mixture was then centrifuged and incubated at 56 °C for 1–3 h. After incubation, the mixture was transferred to a spin column and centrifuged at 11,000× *g* for 1 min. The flow-through solution was discarded. The column was washed by adding 500 µL of buffer BW and spinning at 11,000× *g* for 1 min. After the centrifugation, the flow-through solution was discarded, and the column was washed again using 500 µL of buffer B5 followed by centrifugation at 11,000× *g* for 1 min. The column was again centrifuged at 11,000× *g* for 1 min in order to dry the column. The column was transferred to a 1.5 mL microcentrifuge tube. To elute DNA, 100 µL of BE buffer was added to the column, incubated at room temperature for 1 min, and centrifuged at 11,000× *g* for 1 min. The extracted DNA was stored at −20 °C until use.

### 4.5. LAMP Test

**Figure 1:**
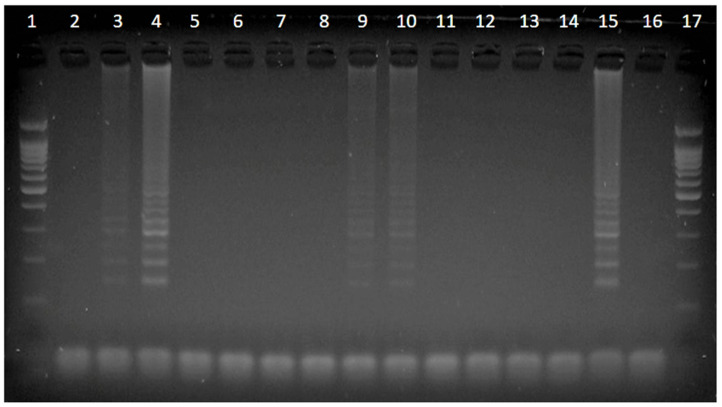
Loop-mediated isothermal amplification (LAMP) products as analyzed on a 2% agarose gel. Lanes 1 and 17: 100 bp molecular ruler; lanes 2–14: samples; lane 15: positive control; lane 16: negative control. Lanes 3, 4, 9, and 10 demonstrate the ladder-like band patterns, which are considered LAMP-positive.

The LAMP reaction was previously performed by mixing 2.6 µL of extracted genomic DNA with 12.4 µL of the reaction solution composed of 10 mM MgSO_4_, 1 M Betaine, 0.6 mM dNTPs, 1.6 µM of inner primers (FIP, BIP), 0.2 µM of outer primers (F3, B3), 1X ThermoPol buffer (20 mM Tris-HCl (pH 8.8), 10 mM KCl, 10 mM (NH_4_)_2_SO_4_, 0.1% Triton X-100), and 8 U of *Bst* DNA polymerase (Lucigen^®^, Lucigen, WI, USA). The primers used in this study were designed by Hong et al. [25] and are listed in Table 4. The reaction was started at 65 °C for 60 min and stopped at 80 °C for 2 min. Then, 5 µL of each of the LAMP products was analyzed using 2% agarose gel electrophoresis and stained with ethidium bromide. Samples were considered positive when a ladder-like band pattern was observed (Figure 1).

### 4.6. Sensitivity and Specificity Estimations

The agreement between the SIT and LAMP test results was assessed using Cohen’s kappa analysis. The agreement was interpreted as a slight, fair, moderate, substantial, and almost perfect agreement when the estimated kappa values were 0–0.20, 0.21–0.40, 0.41–0.60, 0.61–0.80, and over 0.80, respectively [30].

The characteristics of the SIT and LAMP tests for the detection of bTB in milk samples and the true prevalence of the disease were analyzed using Bayesian latent class analysis. The Bayesian model assumes that, for the k populations, the counts (Y_k_) of the different combinations of test results, such as +/+, +/−, −/+, and −/− for the two tests follow a multinomial distribution: Y_k_ | P_qrk_ ~ multinomial (n_k_, {P_qrk_}), where qr is the multinomial cell probability for the two-test outcome combination, and P_qrk_ is a vector of probabilities of observing the individual combinations of test results. Priors for the Se and Sp of the SIT and LAMP tests and priors for bTB prevalence rates were derived from previous studies and experts’ opinions [5,19,20,31,32]. The most likely value (mode) of each parameter was created from the published report means of the central values. The lowest modal was used as a 95% lower limit for the prior distributions to accommodate for the expected large variability in the test performance. These priors were modeled as beta distributions. Prior estimates for the Se and Sp of the SIT test were considered differently for the standard and severe interpretations as shown in Table 5 and Table 6, respectively. The principle for bTB diagnosis of the SIT test is based on the indirect detection of the CMI response, whereas the LAMP test is based on the direct detection of the pathogen DNA. Therefore, a Bayesian model for two conditionally independent tests was implemented in a single population to evaluate the Se and Sp of each test and true disease prevalence [13]. All analyses were performed in JAGS 4.3.0, using the rjags and R2jags packages in R version 4.1.0 [33,34,35]. Posterior distributions were computed after 100,000 iterations of the models, with the first 10,000 discarded as the burn-in phase.

The convergence of the model was checked by a visual inspection of the Gelman–Rubin diagnostic plot using three sample chains with different initial values [36]. Sensitivity analysis of the model was carried out to evaluate the influence of the prior information and the assumption of conditional independence between the SIT and LAMP tests on the posterior estimates [13,14]. These analyses were performed by replacing each prior with a non-informative uniform 0–1 distribution and comparing the DIC between models with and without the covariance term [13].

## 5. Conclusions

This study provides estimates of the characteristics of the currently available test for bTB diagnosis in Thailand (SIT test) and an alternative molecular technique (LAMP test) in milk, using a Bayesian approach. The results emphasize the importance of improving the performance of bTB control and eradication programs. Nevertheless, the low number of positive results limits the estimation of the test performance. Therefore, further studies should be performed in larger dairy cattle populations or areas.

## Figures and Tables

**Table 1:** Cross-classified test results for bovine tuberculosis in dairy cattle from the SIT test and LAMP test.

Test Results		SIT (Standard) ^a^		SIT (Severe) ^b^	
		Positive	Negative	Positive	Negative
**LAMP** ^c^	positive	0	38	6	32
	negative	2	163	9	156

^a^ Single intradermal tuberculin (SIT) test (standard interpretation). ^b^ Single intradermal tuberculin test (severe interpretation). ^c^ Loop-mediated isothermal amplification (LAMP) test.

**Table 2:** Posterior estimates for median and 95% posterior probability interval (PPI) for sensitivity and specificity of the SIT test using standard interpretation and the LAMP test for the diagnosis of bovine tuberculosis, and prevalence of the disease.

Diagnostic Tests	Parameters	Median (%)	95% PPI ^a^ (%)
SIT (standard) ^a^	Sensitivity	63.5	42.1–81.9
	Specificity	99.1	97.1–99.9
LAMP ^b^	Sensitivity	67.2	40.5–88.4
	Specificity	82.0	76.1–87.1
Disease prevalence		3.7	1.4–7.8

^a^ Single intradermal tuberculin (SIT) test (standard interpretation). ^b^ Loop-mediated isothermal amplification (LAMP) test.

**Table 3:** Posterior estimates for median and 95% posterior probability interval (PPI) for sensitivity and specificity of the SIT test using severe interpretation and the LAMP test for the diagnosis of bovine tuberculosis, and prevalence of the disease.

Diagnostic Tests	Parameters	Median (%)	95% PPI ^a^ (%)
SIT (severe) ^a^	Sensitivity	76.1	55.7–90.9
	Specificity	96	92.6–98.5
LAMP ^b^	Sensitivity	68.8	44.8–88.9
	Specificity	84.1	78.2–89.2
Disease prevalence		6.7	3.2–12.1

^a^ Single intradermal tuberculin (SIT) test (severe interpretation). ^b^ Loop-mediated isothermal amplification (LAMP) test.

**Table 4:** Primers for the loop-mediated isothermal amplification (LAMP) test designed by Hong et al. [29].

Primer	DNA Sequence (5′-3′)	Length	Target
F3	CCGGGTGAGGATCCTGAC	18 bp	*esat6*
B3	GACTGGTCGAGCTTCAGC	18 bp	*esat6*
FIP	GAAAGCACCGCGACGGTGTCTTTTCAGACGGATGACCGATTTGG	44 bp	*esat6*
BIP	CGAGGTGTTGGAAGACACGCCTTTTGAACGCCCACACGCCTT	42 bp	*esat6*

**Table 5:** Prior estimates for mode and 95% confidence interval (CI) for sensitivity and specificity values of SIT test (standard interpretation) and LAMP test, and prevalence of disease (%).

Diagnostic Tests	Parameters	Mode	95% CI ^a^
SIT test (standard) ^b^	Sensitivity	71.0	>53.2
	Specificity	98.6	>89.2
LAMP test ^c^	Sensitivity	75.0	>50.0
	Specificity	95.0	>50.0
Disease prevalence		10.0	<20.0

^a^ 95% lower or upper confidence interval bound. ^b^ Single intradermal tuberculin (SIT) test (standard interpretation). ^c^ Loop-mediated isothermal amplification (LAMP) test.

**Table 6:** Prior estimates for mode and 95% confidence interval (CI) for sensitivity and specificity values of SIT test (severe interpretation) and LAMP test, and prevalence of the disease (%).

Diagnostic Tests	Parameters	Mode	95% CI ^a^
SIT test (standard) ^b^	Sensitivity	81.0	>63.0
	Specificity	95.6	>89.2
LAMP test ^c^	Sensitivity	75.0	>50.0
	Specificity	95.0	>50.0
Disease prevalence		10.0	<20.0

^a^ 95% lower or upper confidence interval bound. ^b^ Single intradermal tuberculin (SIT) test (severe interpretation). ^c^ Loop-mediated isothermal amplification (LAMP) test.

## Data Availability

The data presented in this study are available upon request to the corresponding author.
